# Timosaponin AIII Suppresses Hepatocyte Growth Factor-Induced Invasive Activity through Sustained ERK Activation in Breast Cancer MDA-MB-231 Cells

**DOI:** 10.1155/2013/421051

**Published:** 2013-06-25

**Authors:** Chung-Hsin Tsai, Chu-Wen Yang, Jir-You Wang, Yi-Fang Tsai, Ling-Ming Tseng, Kuan-Liang King, Wei-Shone Chen, Jen-Hwey Chiu, Yi-Ming Shyr

**Affiliations:** ^1^Institute of Traditional Medicine, School of Medicine, National Yang-Ming University, No. 155, Section 2, Linong Street, Beitou, Taipei 112, Taiwan; ^2^Division of General Surgery, Department of Surgery, Mackay Memorial Hospital, No. 92, Section 2, Zhongshan N. Road, Taipei 10449, Taiwan; ^3^Department of Microbiology, Soochow University, No. 70, Linhsi Road, Shihlin, Taipei 111, Taiwan; ^4^Department of Orthopedics, Taipei Veterans General Hospital, No. 201, Section 2, Shipai Road, Beitou, Taipei 11217, Taiwan; ^5^Division of General Surgery, Department of Surgery, Taipei Veterans General Hospital, No. 201, Section 2, Shipai Road, Beitou, Taipei 11217, Taiwan; ^6^Department of Surgery, School of Medicine, National Yang-Ming University, No. 155, Section 2, Linong Street, Beitou, Taipei 112, Taiwan; ^7^Division of Colorectal Surgery, Department of Surgery, Taipei Veterans General Hospital, No. 201, Section 2, Shipai Road, Beitou, Taipei 11217, Taiwan; ^8^Experimental Surgery of the Department of Surgery, Taipei Veterans General Hospital, No. 201, Section 2, Shipai Road, Beitou, Taipei 11217, Taiwan; ^9^Division of General Surgery, Department of Surgery, Cheng-Hsin General Hospital, No. 45, Cheng-Hsin Street, Beitou, Taipei 112, Taiwan

## Abstract

*Background*. The aim of this study was to investigate the mechanisms by which Timosaponin AIII (TAIII) is able to inhibit HGF-induced invasion activity in the triple negative breast cancer cell line MDA-MB-231. *Methods*. After pretreatment with different concentrations (10^−6^~10^−8^ M) of TAIII, the cells were treated with hepatocyte growth factor (HGF, 15 ng/mL). At different time intervals after coincubation, various parameters, including the expression of c-Met, ERK, COX2, and MMP-9, which were assessed by Western blotting or by real-time PCR, were analyzed. In addition, invasive activity was also monitored. *Results*. HGF was found to induce c-MET activation and ERK activation, together with increased COX2 protein expression; these changes were followed by a subsequent increase in invasive activity. TAIII was found to suppress HGF-induced invasive activity and *COX2* gene expression in a concentration-dependent manner (10^−6^~10^−8^ M) in parallel with increases in the phosphoforms of c-Met and ERK after TAIII treatment. The mechanisms by which TAIII suppresses HGF-induced invasive activity were demonstrated to include sustained cytoplasmic and nuclear ERK activation; these led to a suppression of nuclear ATF2 activation, which was followed by downregulation of *COX2* and *MMP-9* transcription. *Conclusion*. TAIII suppresses HGF-induced invasive activity in MDA-MB-231 cells via sustained ERK activation.

## 1. Introduction

 Breast cancer is the most common invasive female cancer worldwide, and this is also true for Taiwan [1]. Triple-negative breast cancers (TNBC), a subtype of breast cancer that is characterized by absence of estrogen receptors (ER), progesterone receptors (PR), and a lack of overexpression of human epidermal growth factor receptor 2 (HER2), account for about 15% of all breast cancer cases [2]. These tumors characteristically occur in younger women. They show an aggressive behavior, a high recurrence rate and a high association with distant metastasis of the brain, spinal cord, liver, and lungs. Treatment options are limited since hormonal receptor and HER-2 antagonists, which are typically the approaches used for other breast cancers, are ineffective. The mainstay of the treatment of TNBC is traditional systemic cytotoxic chemotherapy. Recently, an increasing range of biomarkers associated specifically with various subtypes of breast cancer has been identified [3]. Some of these biomarkers are involved in tumor-cell proliferation, survival, and invasiveness, and these may provide a rational basis for the development of molecularly targeted therapies that can treat malignancies such as TNBC. Many factors, including expression of epidermal growth factor (EGF), hepatocyte growth factor (HGF) and insulin-like growth factor, have been demonstrated to correlate well with the invasiveness or metastasis potential of these cancers; these seem to be associated in breast cancer cells with phosphatidylinositol 3-kinase (PI3K) or MAPK (ERK or p38) activation together with COX2 overexpression. The c-Met receptor and/or its ligand HGF are often overexpressed in tumors and have been shown to be associated with a poor prognosis [4, 5]. HGF-induced c-Met-ERK-COX2 signaling is known to be involved in lung cancer invasion [6]. HGF increases COX-2 protein expression by 3-fold over basal levels via both the extracellular signal-regulated kinase 1/2 (ERK) and the p38 pathway that induced prostaglandin E2 (PGE_2_); these seem to enhance lung cancer invasion. Although several multitargeted RTK and SRC family inhibitors have been investigated in clinical trials involving patients with breast cancer, the results were found to be of limited usefulness in TNBC patients [7]. 

In recent decades, there has been a trend whereby the use of complementary and alternative medicines (CAMs) is increasing among oncology patients [8]. Breast cancer patients tend to use CAM more frequently than those with other types of cancer with prevalence rates ranging from 28% to 97% [9]. The most common reason given for patients with breast cancer using CAM is the alleviation of their symptoms after specific treatments, namely hormonal (tamoxifen or aromatase inhibitor) and targeted (herceptin) therapy.

Natural herbal medicines such as *Anemarrhena asphodeloides *have been reported to inhibit the growth of selected cancer cells *in vitro* [10], but the mechanisms by which they bring about such antitumor effects are unclear. *Anemarrhena asphodeloides *is widely used to alleviate insomnia and postmenopausal symptoms, which seem to be common among breast cancer patients who receive chemotherapy in Taiwan. Timosaponin AIII (TAIII) is a steroidal saponin isolated from *Anemarrhena asphodeloides* that has been showed to inhibit cell growth and induce apoptosis in gastric cancer cell lines [11]; furthermore, it has also been shown to induce autophagy preceding mitochondria-mediated apoptosis in HeLa cancer cells [12]. Nevertheless, the effects of TAIII on breast cancer cells, especially on TNBC, remain unclear. Accordingly, the aim of this study was to investigate the effects of TAIII on TNBC MDA-MB-231 cells. In this paper, we demonstrate that TAIII suppresses the HGF-induced invasive activity of MDA-MB-231 cells via sustained ERK activation and that this subsequently suppresses nuclear ATF2 expression, which results in a downregulation of *COX* gene expression.

## 2. Materials and Methods

### 2.1. Cell Line and Reagents

**Figure 1:**
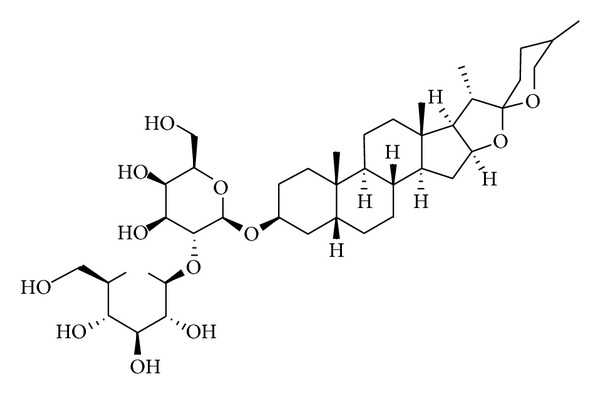
The molecular structure of Timosaponin AIII, C_39_H_64_O_13_. Molecular weight: 740.92.

The MDA-MB-231 (ER^−^, HER2 low) breast cancer cell line was obtained from American Type Culture Collection (ATCC, Manassas, VA, USA). MDA-MB-231 cells were maintained in F12 MEM (NO. 12400-024, Gibco, NY, USA) supplemented with 10% FBS, 2 mM L-glutamine, and penicillin/streptomycin; they were cultured at 37°C in a humidified atmosphere containing 5% CO_2_. Timosaponin AIII (TAIII, Figure 1), a pure compound derived from *Anemarrhena asphodeloidea* Bunge, was obtained commercially (206-13391, Wako Pure Chemical Industries, Ltd. Osaka, Japan).

### 2.2. Cytotoxicity Assay by MTT Assay

MDA-MB-231 cells at a density of 3 × 10^5^ cells were seeded in each well of a 6-well plate overnight. This was followed by treatment with various concentrations of TAIII (10^−6^, 10^−7^, and 10^−8 ^M) for different time intervals (1 day, 2 days, and 3 days). The cells were washed twice with phosphate buffered saline (PBS) pH 7.4. For the MTT assay, both the treated and untreated cells were incubated with 100 *μ*L MTT (tetrazolium compounds) for 4 h and lysed with 100 *μ*L DMSO. After this the optical density was read using an ELISA reader at a wavelength of 570 nm.

### 2.3. Cell Invasion Assay

The *in vitro* invasiveness of MDA-MB-231 cells was assessed using a modified Boyden chamber (BW200s, Neuroprobe, MD, USA) assay [13]. A total of 2 × 10^5^ cells were suspended in 150 *μ*L of serum-free medium and placed in the upper compartment of the Boyden chamber containing a membrane (8 *μ*m pore size, 13 mm diameter, K80SH01300, GE Water & Process Technologies, USA) that had been coated with 50 *μ*L Matrigel (NO. 356231, BD Biosciences Bedford, MA, USA). The lower compartment of the chamber was then filled with 230 *μ*L of 0.5% FBS containing medium with or without drug treatment (using 10% FBS as the positive control). The cells were then allowed to migrate for 8 h. After incubation the cells on the reverse side of the membrane were fixed and stained with Mayer's haematoxylin; finally, the cells were photographed.

### 2.4. Cell Migration Assay (Wound Healing Assay)

*In vitro* cell migration of MDA-MB-231 cells was measured using a cell culture insert (NO. 80209, Ibidi, Munich, Germany). In brief, 5 × 10^4^ cells were seeded together with an insert on a 3.5 cm Petri dish overnight. After washing the cells with PBS, the insert was removed and the cells were cultured with or without drug treatment. After 24 h of incubation, the cells were examined by light microscopy and photographed.

### 2.5. Western Blotting Analysis

Cultured cells were lysed in a buffer containing 150 mM KCl, 10 mM Tris pH 7.4, 1% Triton X-100, and protease inhibitors cocktail (Complete Mini; Roche, Mannheim, Germany). The protein concentrations in cell homogenates were measured using Bradford's method [14]. Protein samples (50 mg of protein) were separated by 10% SDS-PAGE and then transferred to a nitrocellulose membrane (Hybond-C; Amersham Biosciences, NJ, USA). The membrane was blocked with 5% bovine serum albumin and probed with specific primary antibodies. Antibodies targeting the following proteins were used: p-c-Met (phospho Y1349, # ab47606, Abcam), c-Met (EP1454Y, # ab51067, Abcam), p-ERK (Thr202/Tyr204, #4370, Cell Signaling), ERK (#4695, Cell Signaling), COX-2 (#160112, Cayman), *α*-TUBULIN (Sigma), *β*-ACTIN (Sigma), ATF2 (#9226, Cell Signaling), p-ATF2 (#9221, Cell Signaling), and GAPDH (sc-25778, Santa Cruz Biotechnology, Inc.). Immunoreactivity was detected using a luminescent image analyzer (FujiFilm, LAS-1000).

### 2.6. Fractionation of Nucleus and Cytosol

Nuclear and cytosol extracts of the cultured cells were obtained as described previously [15]. In brief, the cells were harvested and washed with cold PBS. Next, the cells were re-uspended in RIPA lysis buffer (including 5 mM PIPES pH 8.0, 85 mM KCl, 0.5% NP-40, and proteinase and phosphatase inhibitors) and lysed using a Dounce tissue grinder; the lysates were then incubated on ice for 10 min. At this point, the cytosol and nucleus fractions were separated by centrifugation at 3000 rpm and 4°C for 10 min. RIPA buffer was used to extract the proteins present in the nucleus fraction.

### 2.7. Total RNA Extraction and Reverse Transcription PCR

Total RNA was isolated using a modified single-step guanidinium thiocyanate method (TRI REAGENT, T-9424, Sigma Chem. Co., St. Louis, MO, USA) [16]. Complementary DNA (cDNA) was prepared from the total RNA using a First Strand cDNA Synthesis Kit (Invitrogen, CA, USA). The *de novo* gene synthesis changes across the various treatment groups were detected by reverse transcriptase-polymerase chain reaction (RT-PCR). The gene expression of metastasis-related proteins, including *COX2*, and *MMP-9*, was elucidated using commercially available primers. The paired primers used for COX2 were forward 5′-GCTGAGCCATACAGCAAATCC-3′ and reverse 5′-GGGAGTCGGGCAATCATCAG-3′. The paired primers used for MMP-9 were forward 5′-CACTGTCCACCCCTCAGAGC-3′ and reverse 5′-GCCACTTGTCGGCGATAAGG-3′. The paired primers used for *β*-actin were forward 5′-ACCCACACTGTGCCCATCTAC-3′ and reverse 5′-TCGGTGAGGATCTTCATGAGGTA-3′. Possible contamination of any PCR component was excluded by performing a PCR reaction with these components in the absence of the RT product for each set of experiments (nontemplate control, NTC). Quantification of the RNA transcripts was carried out as described in a previous method with some modification. For statistical comparison, the relative expression of the mRNA of specific genes was normalized against the amount of GAPD in the same RNA extract. All samples were analyzed in triplicate.

### 2.8. Zymography to Measure MMP-9 Protein Levels

The enzymatic activity of MMP-9 was determined by gel zymography. Briefly, cells were seeded and allowed to grow to confluence for 24 h and then maintained in serum-free (SF) medium. Conditioned medium was then collected at 24 h after SF incubation. This was mixed with nonreducing sample buffer and subjected to electrophoresis on a 10% polyacrylamide gel containing 0.1% (w/v) gelatin. Next, the gel was washed with washing buffer (2.5% Triton X-100, 50 mmol/L Tris-HCl, pH 7.5) and incubated at 37°C for 24 h in 50 mmol/L Tris-HCl (pH 7.5), 150 mmol/L NaCl, 5 mmol/L CaCl_2_, 1 mmol/L ZnCl_2_, and 40 mmol/L NaN_3_. Finally, the gel was stained with 0.25% (w/v) Coomassie brilliant blue in 45% (v/v) methanol and 1% (v/v) acetic acid, examined, and photographed.

### 2.9. Statistical Analysis

Data are expressed as the mean ± SEM. Differences between groups at the various time point were identified by one-way ANOVA followed by the Dunnett's *post hoc* test. A *P* value of <0.05 was considered statistically significant when compared to the vehicle or no treatment group.

## 3. Results

### 3.1. Effects of TAIII on the HGF-Induced Invasive activity of MDA-MB-231 Cells

**Figure 2:**
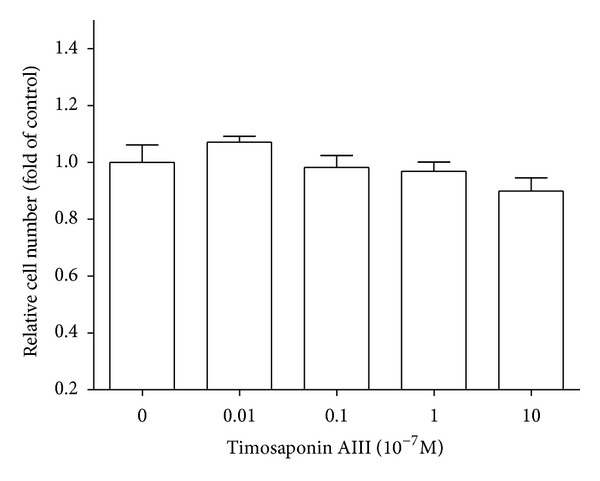
Effects of Timosaponin AIII on cytotoxicity of MDA-MB-231. The cell toxicity was evaluated by MTT assay 48 h after TAIII administration (10^−9^ to 10^−6 ^M) as described in Section 2.

**Figure 3:**
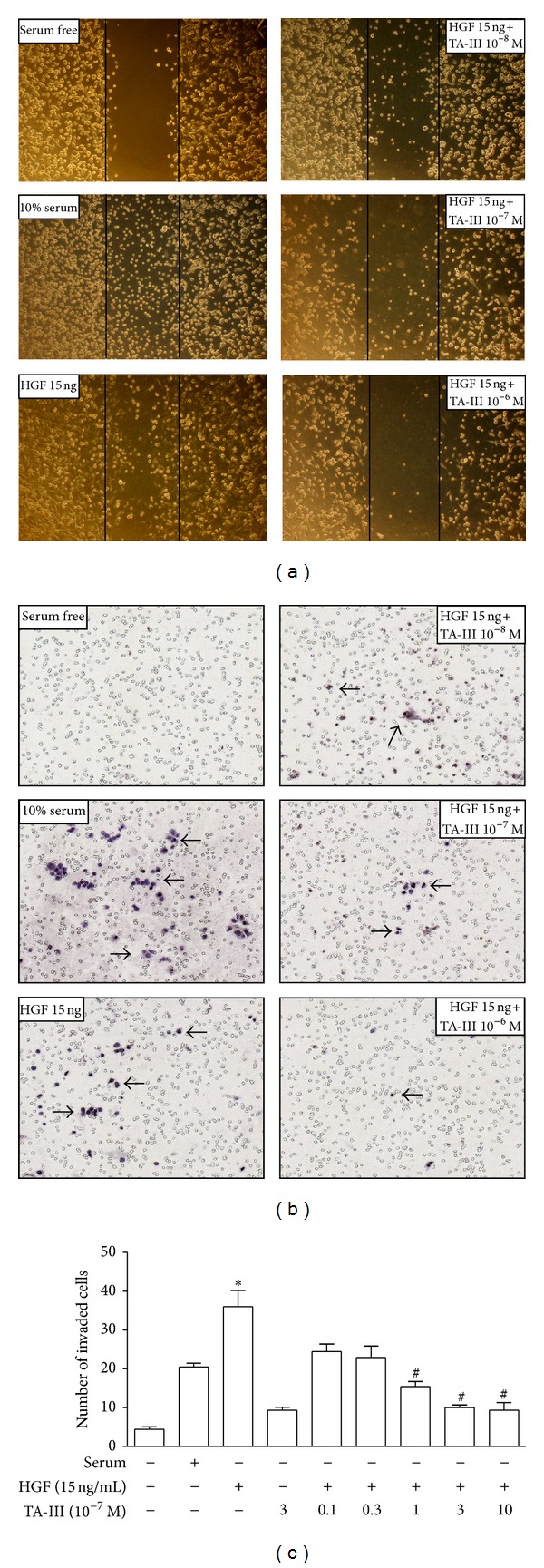
Effects of timosaponin AIII on HGF-induced invasive activity in MDA-MB-231 cells. The migration assay (a) was used with a cell culture insert, and *in vitro* invasiveness of MDA-MB-231 cells was assessed using a modified Boyden chamber (b, c) as described in Section 2. TAIII concentration dependently (10^−8^~10^−6 ^M) suppressed HGF-induced invasive activity, either by migration assay or by invasion assay. The data obtained from invasion assay was quantified (c). The arrow indicates the invaded cells on the membrane of Boyden chamber. *P < 0.05 compared to serum free; ^#^ *P* < 0.05 compared to HGF (15 ng/mL) alone-treat group (one-way ANOVA, followed by Dunnett's *t* test).

Having been shown to exhibit little cytotoxicity (Figure 2), it was found that TAIII inhibited the HGF-induced invasive activity of MDA-MB-231 cells in concentration-dependent manner (10^−6^~10^−8 ^M), both by migration assay (Figure 3(a)) and by invasion assay (Figures 3(b) and 3(c)).

### 3.2. Effects of TAIII on HGF-Induced *COX2* Gene Expression in MDA-MB-231 Cells

**Figure 4:**
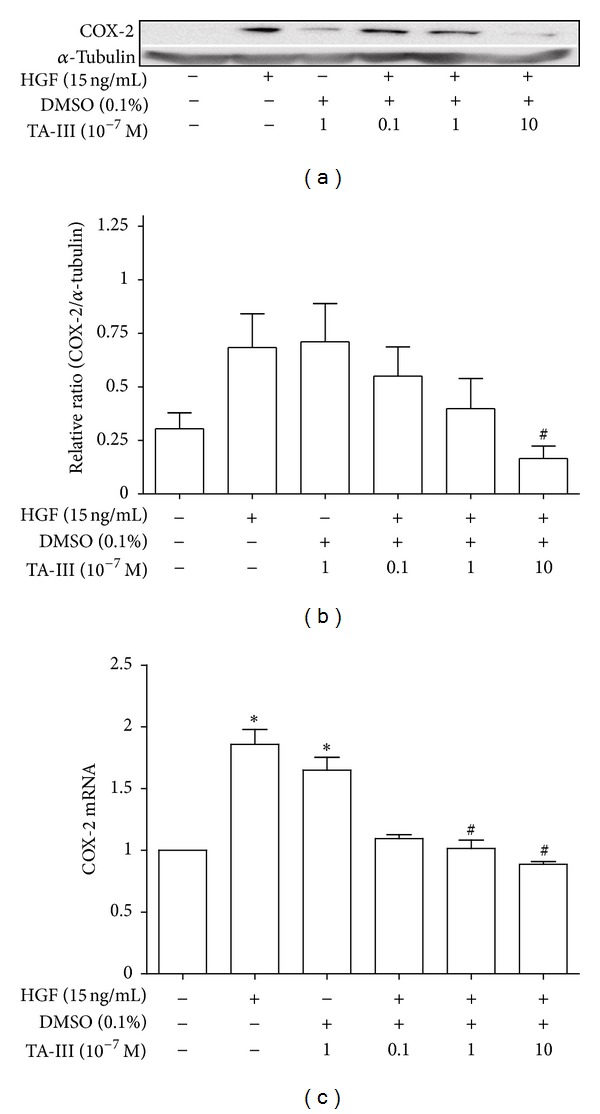
Effects of Timosaponin AIII on HGF-induced *COX2* gene expression in MDA-MB-231 cells. TAIII concentration dependently (10^−8^~10^−6 ^M) inhibited HGF-induced *COX2* gene expression by Western blot (a, b) and by real-time PCR (c) 24 h and 12 h after drug treatment in MDA-MB-231 cells, respectively. *P < 0.05 compared to serum free; ^#^ *P* < 0.05 compared to HGF (15 ng/mL) alone-treat group (one-way ANOVA, followed by Dunnett's *t* test).

In order to elucidate the role of COX2 protein in the TAIII suppression of HGF-induced invasive activity, cultured cells were cotreated with TAIII and HGF, and this was followed by Western blot analysis and real-time PCR to assess *COX2* gene expression. The results showed that TAIII inhibits the HGF-induced *COX2* gene expression of MDA-MB-231 cells as measured by Western blotting (Figures 4(a) and 4(b)) and by real-time PCR (Figure 4(c)); this occurred in a concentration-dependent manner (10^−6^~10^−8 ^M) at 12 h and at 24 h after drug treatment.

### 3.3. The ERK-Related COX2-Mediated Pathway Plays a Role in How TAIII Affects HGF-Induced Invasive Activity in MDA-MB-231 Cells

**Figure 5:**
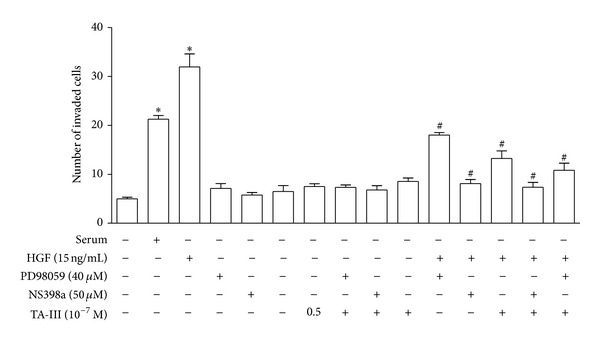
Effects of inhibitors on Timosaponin AIII suppressed HGF-induced invasive activity. The *in vitro* invasiveness of MDA-MB-231 cells was assessed using a modified Boyden chamber as described in Section 2. Timosaponin AIII-inhibited HGF-induced invasive activity could be completely blocked by COX2 inhibitor (NS398) and partially blocked by ERK inhibitor (PD98059).

Figure 5 showed that the TAIII inhibition of HGF-induced invasive activity is able to be completely blocked by a COX2 inhibitor (NS398) and is also partially blocked by an ERK inhibitor (PD98059). The results suggested the ERK-related COX2-mediated pathway plays a role that in how TAIII affects HGF-induced invasive activity in MDA-MB-231 cells.

### 3.4. TAIII Enhances HGH-Induced c-Met Phosphorylation and Sustains ERK Phosphorylation in MDA-MB-231 Cells

**Figure 6:**
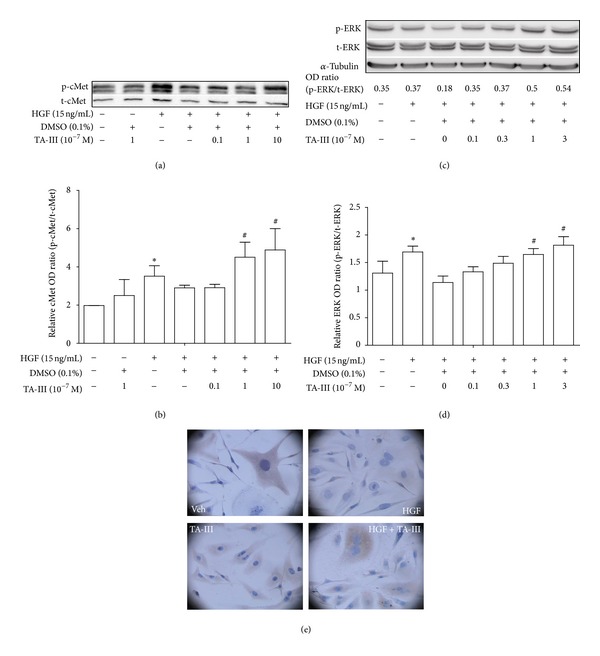
Effects of Timosaponin AIII on c-Met and ERK phosphorylation. c-Met (a, b) and ERK (c, d) proteins expression was analyzed at 5 min and 2 h after HGF treatment as described in Section 2. Pretreatment of TAIII (10^−8^~10^−6 ^M) enhanced the phosphorylation of these proteins in a concentration-dependent manner. By immunohistochemistry (e), sustained ERK phosphorylation could be demonstrated even 24 h after TAIII and HGF cotreatment.

When analyzed by Western blotting, it was found that HGF-induced the phosphorylation of the c-Met (Figures 6(a) and 6(b)) and ERK (Figures 6(c) and 6(d)) proteins, which occurred at 5 min and 2 h after HGF treatment, respectively. However, pretreatment with TAIII (10^−6^~10^−8 ^M) enhanced the phosphorylation of these proteins in a concentration-dependent manner. Using immunohistochemistry, sustained ERK phosphorylation could be demonstrated even 24 h after TAIII and HGF cotreatment (Figure 6(e)).

### 3.5. TAIII Changes the Kinetics of HGH-Induced Nuclear ERK Phosphorylation in MDA-MB-231 Cells

**Figure 7:**
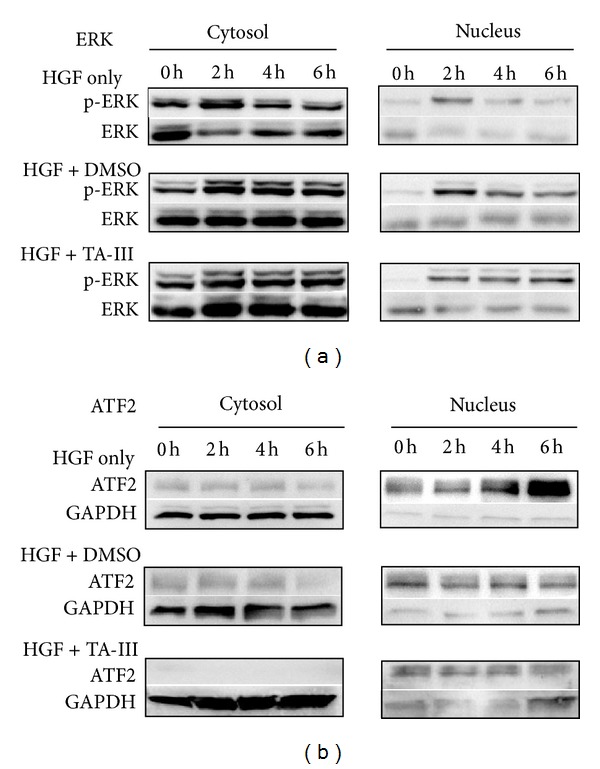
Effects of Timosaponin AIII on the kinetics of ERK phosphorylation and ATF2 expression in MDA-MB-231 cells. The nuclear extraction proteins induced by HGF were analyzed by Western blot as described in Section 2. The results showed a kinetic increased expression of p-ERK (a) and ATF2 (b) with the peak at 2 h and 6 h, respectively. TAIII (10^−7^ M) changed the kinetics of p-ERK induced by HGF with the peak at 6 h, while suppressed HGF-induced nuclear ATF2 expression.

The proteins extracted from the nucleus after the cells were induced with HGF showed an increased expression of p-ERK and ATF2 that peaked at 2 h and 6 h, respectively. TAIII (10^−7^ M) changed the kinetics of p-ERK induction by HGF, and the peak was delayed 6 h (Figure 7(a)), while at the same time there was a suppression of HGF-induced nuclear ATF2 expression (Figure 7(b)).

### 3.6. Effects of TAIII on HGF-Induced *MMP-9* Gene Expression in MDA-MB-231 Cells

**Figure 8:**
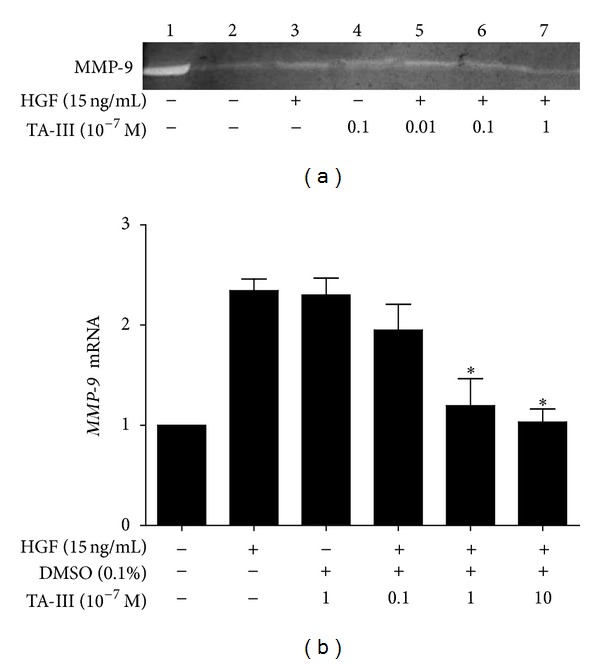
Effects of Timosaponin AIII on *MMP9* gene expression in MDA-MB-231 cells. MMP9 expression was analyzed by zymography (a) and real-time PCR (b) as described in Section 2. The results showed that that TAIII concentration dependently (10^−8^~10^−6 ^M) inhibits HGF-induced *MMP-9* gene expression by zymography and by real-time PCR in MDA-MB-231 cells.

In order to elucidate the role of other metastasis-related proteins such as MMP-9, zymography and real-time PCR were performed. The results showed that TAIII inhibited HGF-induced *MMP-9* gene expression in MDA-MB-231 cells as measured by zymography (Figure 8(a)) and by real-time PCR (Figure 8(b)) in a concentration-dependent manner (10^−6^~10^−8^ M) at 12 h and 24 h after drug treatment, respectively.

### 3.7. TA-III Induces the Generation of Intracellular Oxygen Reactive Species (ROS) in MDA-MB-231 Cells

**Figure 9:**
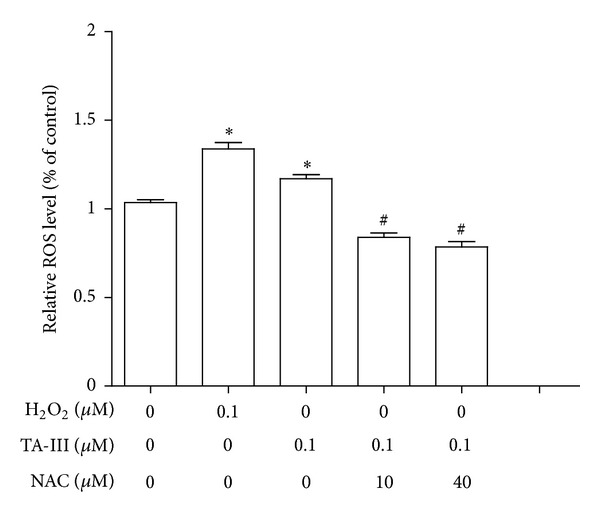
Effects of Timosaponin AIII on intracellular reactive oxygen species (ROS) production in MDA-MB-231 cells. The intracellular ROS was measured using H_2_O_2_ as a positive control described in Section 2. Pretreatment of N-acetlycystein (NAC) (10 *μ*M and 40 *μ*M) attenuated TAIII induced-ROS production. *P < 0.05 compared to negative control group. ^#^ *P* < 0.05 compared to TAIII-alone group. (One-way ANOVA, followed by Dunnett's *t* test).

Since ROS is an important factor that induces the phosphorylation of ERK protein, N-acetylcysteine (NAC) was used to scavenge intracellular ROS. The results showed that pretreatment with NAC (10 *μ*M and 40 *μ*M) attenuated the TAIII-induced increase in intracellular ROS level (Figure 9).

## 4. Discussion

In the present study, we have shown that Timosaponin AIII inhibits HGF-induced invasive activity in a concentration-dependent manner. It is postulated that the mechanism involved is sustained ERK activation, which then suppresses nuclear ATF2 expression and downregulates *COX* gene expression. Since TAIII at the concentration 10^−7^ *μ*M, which is able to suppress invasive activity, does not show cell cytotoxicity, this supports the hypothesis that the mechanism by which TAIII causes a cytotoxic effect on MDA-MB-231 cells is different from that by which it inhibits HGF-induced invasiveness. This is supported by the fact that there is only a limited inhibition of cell growth when an MTT assay is carried out in the presence of TAIII. Thus, TAIII seems to be able to inhibit TNBC metastasis through an inhibition of invasion and/or migration. Actually, the changes to cMet phosphorylation, which is the initial signal related to HGF, are not modified by TAIII treatment at the concentrations considered effective for HGF-induced cMet phosphorylation. Therefore, direct modification of HGF by TAIII or the blockade of HGF-induced cMet signaling by TAIII can be ruled out.

Previously, induction of COX-2 through the activation of the ERK2 signal pathway by HGF has been demonstrated in gastric cancer [17, 18]. Furthermore, COX-2 has been postulated to play an important role in tumor metastasis across many tumor types, including lung cancer [19]. Targeting of COX-2 is under investigation as both a cancer prevention measure and as a cancer treatment. Inhibition of COX-2 limits the production of prostaglandins, which are well known to stimulate cell proliferation, induce invasiveness, and mediate angiogenesis [20, 21]. In the present study, we found that TAIII inhibited TNBC cell invasion through the HGF-induced c-Met-ERK-COX2 signaling pathway. This mechanism is different from that proposed for the gastric cancer cell line, which has been proposed to involve the apoptosis machinery, and is also different to that described for HeLa cancer cells, which has been proposed to involve autophagy.

Several lines of evidence support the idea that extracellular signal-related kinase (ERK) plays an important role in cell signaling. Although this has been accepted as a key player in protecting cells against death by apoptosis, it is now clear that ERK can also be directly linked to cell death signaling [22]. Conventionally, HGF induces p-ERK expression in the nucleus peaks at 2 h and then gradually decreases at 4 h and 6 h. Simultaneously, ERK activation induces the expression of many genes, including its own regulators, the DUSPs (ERK-specific phosphatases). Thus, ERK activity rapidly reaches a steady state and its death-promoting activity remains at low levels resulting in a progressive accumulation of death-promoting factors that eventually reach a level that induces cell death [23]. The activation of ERK might also transiently increase death-promoting activities of other death stimuli, such as chemotherapeutic agents. Sustained cytoplasmic ERK activity might promote senescence or autophagy, whereas sustained nuclear sequestration of ERK activity seems to trigger apoptosis. It has been documented that cadmium induces cell death by sustained ERK activation via the oxidative response of cells and the production of ROS. Any agents that provoke a sustained activation of ERK, such as ROS, should be able to inhibit DUSP, thus inducing cell apoptosis. In the present study, we found that TAIII affected the kinetics of the HGF-induced phosphorylation of ERK; specifically, p-ERK expression remained high in nucleus even at 6 h after TAIII treatment, while the level of p-ERK in the HGF alone group had decreased. Similar results were also observed using immunohistochemical staining. Both immunohistochemistry and Western blot analysis showed that our results are compatible with sustained ERK activation. Furthermore, we used exogenous H_2_O_2_ as a positive control to produce intracellular ROS in MDA-MB-231 cells. It is noteworthy that the level of ROS produced by TAIII treatment is able to be deceased by pretreatment with a ROS scavenger (N-acetylcysteine, NAC), which indicates that TAIII alone (without HGF treatment) is able to induce oxidative stress, an important factor associated with the induction of ERE activation. Interestingly, the Ras/Raf/RK pathway plays a critical role in promoting several forms of cell death in response to numerous stress stimuli both *in vitro*, with various cellular models, and *in vivo*. A common hallmark of this response is the sustained activation of ERK, which contrasts with the transient nature of ERK stimulation found in situations where ERK regulates other cell fates [22]. A recent study has demonstrated that TAIII at the high concentrations (*μ*M) is preferentially cytotoxic with respect to tumor cells via an inhibition of mTOR and an induction of ER stress [24], which is in agreement with our findings.

The activity of ERK1/2 and p38 in cells after HGF treatment is linked to the downstream activation of three transcription factors that are known to recognize COX-2 promoter elements, namely, AP-1, C/EBP, and CREB [25]. Both ERK1/2 and P38 seem to participate in and are necessary for effective transcription factor activation of COX-2. Activating transcription factor 2 (ATF2), one of the CREB family, is commonly found at metastatic sites [26] and has been shown to be involved in melanoma development, especially in the HGF/SF transgenic mouse. ATF2 expression has also been reported to be associated with shorter survival of patients with mammary carcinomas [27]. Clinical investigation suggests that patients that show high ATF2 expression have a significantly shorter overall survival rate. This is in agreement with our finding that a low ATF2 level downregulates COX2 transcription and hence suppresses cancer cell invasion. However, whether ATF2 elicits suppressor or oncogenic activities is not well understood [28]. A previous study of more than 500 melanoma patients has revealed that strong cytoplasmic ATF2 expression is associated with primary specimens and better survival, whereas strong nuclear ATF2 expression is associated with metastatic specimens and poor survival [29]. In our results obtained using nuclear and cytosolic extracts, it was found that a decrease in nuclear ATF2 expression is correlated with a suppression of cell invasiveness, which supports the above-mentioned observations.

In addition to ERK-COX2 signaling, other enzymes like matrix metalloproteinases (MMPs) are also involved in the various steps of metastasis development [30]. An increased expression of MMPs, particularly MMP-2 (gelatinase A; 72-kDa type IV collagenase) and MMP-9 (gelatinase B; 92-kDa type IV collagenase), has been correlated extensively with the malignancy of tumors and poor survival among patients, especially those with breast cancer [31, 32]. The fact that TAIII inhibits HGF-induced *MMP-9* gene expression in a concentration-dependent manner (10^−6^~10^−8 ^M) suggests that ERK-COX2 signaling is not the only mechanism by which TAIII suppresses HGF-induced invasive activity.

In summary, the role of complementary and alternative medicine (CAM) continues to evolve and has become a significant part of the daily lifestyle and treatment regimens of cancer patients. Chinese medicine has been practiced for more than thousand years and often uses herbs to achieve its goal of counteracting the side effect of cancer treatment of boosting the immune system, of alleviating the cancer symptoms, and of perhaps tackling the cancer directly [33]. A previous investigation showed that low concentrations of TAIII are able to induce cell death in tumor cells but not in normal cells. The present study has shown that TAIII at low concentrations is able to suppress HGF-induced invasive activity. In summary, our results suggest that TAIII could be an attractive candidate for the development as a cancer therapeutic agent.
